# Early life vitamin D and neurocognitive abilities at age 6–8 years: a randomized clinical trial and observational analysis

**DOI:** 10.1007/s00787-025-02891-7

**Published:** 2025-10-10

**Authors:** Vilja Seppälä, Samuel Sandboge, Elisa Holmlund-Suila, Helena Hauta-alus, Sakari Lintula, Eero Kajantie, Outi Mäkitie, Sture Andersson, Katri Räikkönen, Kati Heinonen

**Affiliations:** 1 Welfare Sciences, Faculty of Social Sciences, Tampere University, Tampere, Finland; 2 Population Health Unit, Finnish Institute for Health and Welfare, Helsinki and Oulu, Finland; 3 Pediatric Research Center, Children’s Hospital, Helsinki University Hospital and University of Helsinki, Helsinki, Finland; 4 Research Program for Clinical and Molecular Metabolism (CAMM), Faculty of Medicine, University of Helsinki, Helsinki, Finland; 5 Clinical Medicine Research Unit, University of Oulu, Oulu, Finland; 6 Folkhälsan Institute of Genetics, Helsinki, Finland; 7 Department of Psychology and Logopedics, Faculty of Medicine, University of Helsinki, Helsinki, Finland; 8 HUS Helsinki University Hospital, University of Helsinki, Helsinki, Finland; 9 Department of Clinical and Molecular Medicine, Norwegian University of Science and Technology, Trondheim, Norway; 10 Department of Molecular Medicine and Surgery, Karolinska Institutet, and Clinical Genetics, Karolinska University Hospital, Stockholm, Sweden; 11 Department of Obstetrics and Gynecology, Helsinki University Hospital, University of Helsinki, Helsinki, Finland

**Keywords:** Vitamin d, Neurocognitive abilities, 25(OH)D, Children

## Abstract

Vitamin D is suggested to impact neurodevelopment, yet the current evidence is conflicting. We tested if vitamin D_3_ supplementation during the first two years of life, maternal 25-hydroxy-vitamin D (25(OH)D) concentration during pregnancy, and child’s 25(OH)D concentrations at 12 and 24 months are associated with neurocognitive abilities in 6.6-8.9-year-old children. Participants were from a Finnish vitamin D double-blind randomized clinical trial and classified themselves white. Children received either vitamin D_3_ 400-IU (*n* = 189) or 1200-IU (*n* = 209) daily until 24 months of age. Serum 25(OH)D was analyzed from mothers at mean of 11.2 weeks of gestation, and from children at 12.0 and 23.9 months (25(OH)D, mean (SD) = 83.8 (21.7), 102.3 (30.1), 105.5 (28.6) nmol/L, respectively). Total Intelligence quotient (IQ) score was assessed with Wechsler Intelligence Scale for Children (WISC-IV) and executive functioning with A Developmental Neuropsychological Assessment (NEPSY-II) and parent-rated Behavior Rating Inventory of Executive Function (BRIEF) questionnaire. Neurocognitive abilities at age 6–8 years did not differ between the supplementation groups or according to 25(OH)D concentration at 12 or 24 months (total IQ: β = 1.14, 95% CI -1.69;3.97, *p* = 0.43, executive functioning, NEPSY-II: β =-0.07, 95% CI -0.14;0.28, *p* = 0.49, executive functioning, BRIEF: β =-0.42, 95% CI -5.13;4.28, *p* = 0.86). Maternal 25(OH)D was not associated with executive functioning. However, both lower and higher maternal 25(OH)D concentrations during pregnancy were associated with child’s lower total IQ scores (vertex at 76.6 nmol/L, quadratic estimate *p* < 0.001) supporting the hypothesis of the role of vitamin D during fetal development. The study is registered with ClinicalTrials.gov (NCT01723852, 2012-11-07 and NCT04302987, 2020-03-06).

**Supplementary Information:**

The online version contains supplementary material available at 10.1007/s00787-025-02891-7.

## Introduction

Vitamin D, clinically measured as serum 25-hydroxyvitamin D (25(OH)D) concentration, is a neuroactive steroid hormone with a multifaceted function, including direct effects on brain development [1]. Vitamin D is needed for normal brain development and alterations in 25(OH)D concentration have been linked to structural and functional brain abnormalities in animal studies [1, 2] and poorer neurocognitive outcomes in human studies [3–5].

Pregnancy and early childhood are critical developmental periods for neurodevelopment [6, 7]. In recent meta-analyses of observational studies, low vitamin D levels during pregnancy were associated with poorer general cognitive ability test scores during early childhood with small to moderate effect sizes [8, 9]. However, significant heterogeneity was reported between the included 9 studies [8]– [9]. Lower maternal vitamin D level is also suggested to be related to inferior childhood executive functioning [10]. The association between early childhood vitamin D levels and later neurocognitive abilities has been studied less. One study reported a cross-sectional association between low vitamin D levels in childhood and poorer general cognitive abilities in typically developing children [11], yet others have not found significant association either with general cognitive abilities or executive functioning [12–14]. Despite the lack of systematic significant findings in observational studies focusing on vitamin D levels during childhood, randomized trial studies targeting children between 6 and 14 years of age and lasting at maximum six months have reported an enhancement in neurocognitive abilities following higher vitamin D supplementation [15–18].

There are many possible factors explaining the inconsistencies in the previous results of associations between vitamin D and neurocognitive abilities. These include high variability in age at the measurement point and in methods used to assess vitamin D or neurocognitive abilities. Thus, it is still too early to conclude on effects of early life vitamin D on neurocognitive abilities [5, 19, 20]. Furthermore, there is a lack of randomized vitamin D supplementation trials targeting early childhood and systematic long-lasting follow-ups [20, 21]. In addition, to our knowledge only two of all the childhood randomized trials focus on typically developing children [16, 17], and they have relatively small sample sizes (119 and 50).

The current study reports a secondary analysis of vitamin D intervention in infants (VIDI) trial, in which children were randomized to receive either 1200-IU or 400-IU dose of vitamin D_3_ supplementation daily from two weeks to 2 years of age. Our earlier study did not find effects of vitamin D supplementation during early childhood on parent-reported developmental milestones at the age of one or two years [22]. In the current study, we focus on neurocognitive abilities in early school age (age 6–8 years) to address possible sleeper-effect of the intervention. Neurocognitive abilities continue to develop significantly during early childhood and school age [23], and the beginning of formal schooling places increased demands on cognitive abilities and executive functioning. In addition to reporting potential intervention effects on neurocognitive abilities measured as general cognitive abilities and executive functioning using performance-based and parent-reported measurements at 6–8 years of age, we also test the longitudinal association with maternal 25(OH)D concentration during pregnancy and child’s 25(OH)D concentration at 12 and 24 months of age.

## Methods

### Participants

**Fig. 1:**
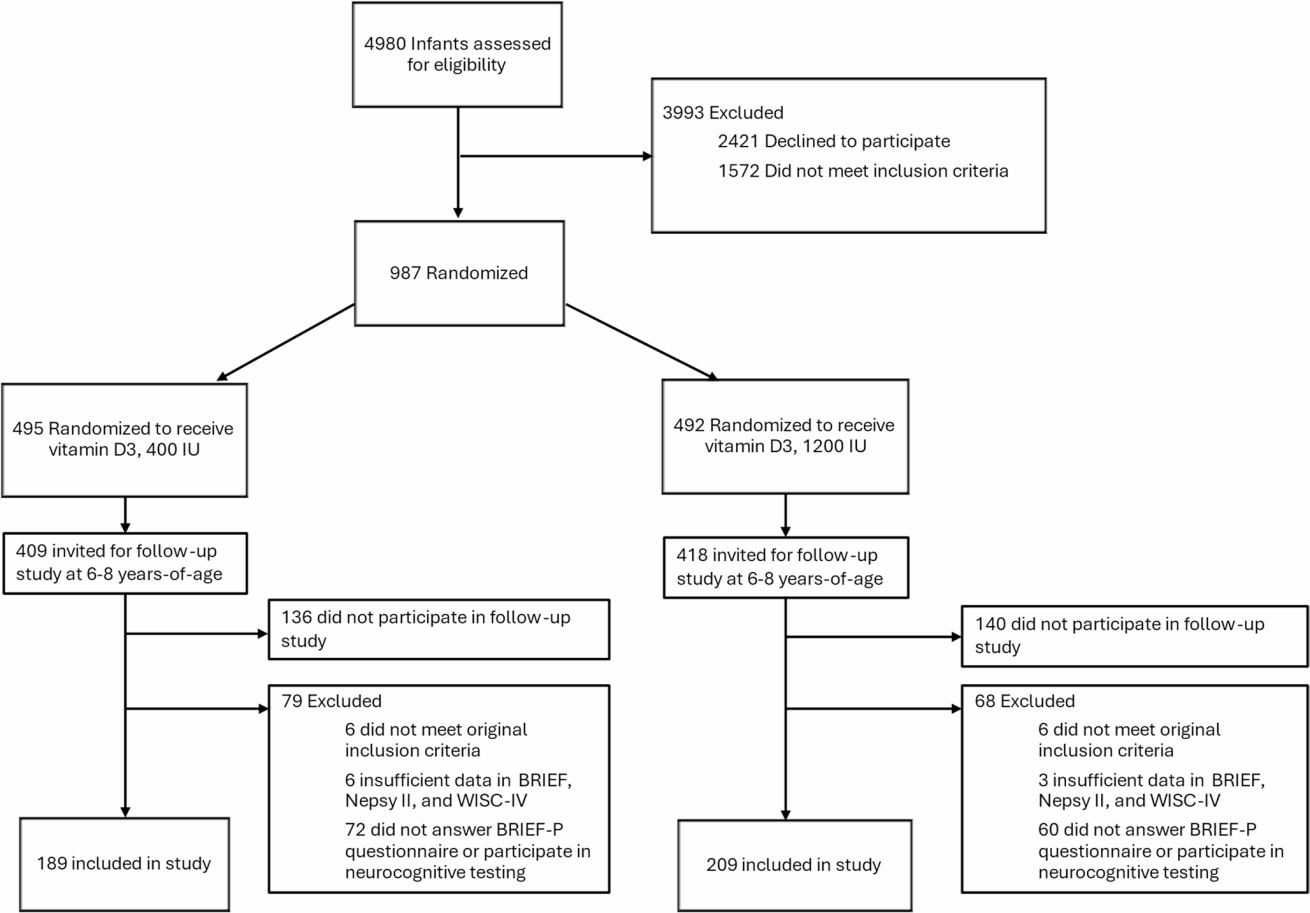
Flowchart of study enrollment, allocation, and follow-up

The study comprised 398 children from the VIDI trial (Fig. 1). VIDI is a double-blind, randomized clinical trial studying vitamin D intervention in healthy term-born children [24]. The 987 (492 female) infants and their families were recruited from Kätilöopisto Maternity Hospital in Helsinki, Finland between January 1, 2013, and June 30, 2014. All mothers were residing in Finland and classified themselves as white. Children’s parents signed informed consent forms at baseline and at the 6–8–year follow-up and children gave written assent to participate at the 6–8–year follow-up. The study was approved by the ethics committee at the Hospital District of Helsinki and Uusimaa and performed in line with the principles of the Declaration of Helsinki. The study is registered with ClinicalTrials.gov (NCT01723852 [VIDI] and NCT04302987 [VIDI2]). The study follows the Consolidated Standards of Reporting Trials (CONSORT) reporting guideline.

### Procedure

Infants were randomized on a 1:1 basis to receive either 400-IU or 1200-IU of vitamin D_3_ supplementation daily from age 2 weeks to 24 months [24]. Details of the vitamin D_3_ supplementation are described in Online Resource Appendix 1, and elsewhere [24]. Serum samples used in current study were collected when infants were at mean of 12.0 (SD = 0.3) and 23.9 (SD = 0.4) months old. Parents filled in questionnaires on health and family demographics at birth and at 12-month, 24-month and 6–8-year follow-up visits. Hospital records were used to collect prenatal information on the mother and child. Maternal serum samples were collected during routine maternity clinic visits at mean of 11.18 (SD = 1.93) gestational weeks and stored in the Finnish Maternity Cohort serum bank organized by the Finnish Institute for Health and Welfare.

#### Follow-up at 6 to 8 years

The follow-up study began in November 2019. We invited 817 families who remained in the original VIDI study through the intervention at age 2 years and had an available home address. Out of those invited, 546 families (55.3% of the original participants) participated the follow-up study, with 456 families completing at least one psychological or cognitive online survey and 300 children participating in neurocognitive testing between March 2020 and May 2022. The personnel conducting the follow-up were blinded to the intervention group.

Of the 398 children (Fig. 1) of this secondary analysis of randomized clinical trial, 189 belonged to the 400-IU D_3_ group and 209 to the 1200-IU D_3_ group. Performance-based Total Intelligence quotient (IQ) score was assessed from 278, executive functioning from 210, and parent-rated executive functioning from 320 children. Comparison of participants against the attrition group are shown in Online Resource Table 1. In the analytic sample, parents had higher educational level, parity was lower, and mothers were older, smoked less, breastfed longer and had higher 25(OH)D concentrations (all p-values < 0.05) than in the attrition group. The children in the analytic sample had higher 25(OH)D concentrations at 12 and 24 months (p-values < 0.01) and were more often born in summer and autumn than the children in the attrition group (*p* < 0.01). Attrition or characteristics for nonparticipants did not differ between intervention groups (all p-values > 0.05, Online Resource Table 2) except for maternal age and parity, which were higher among nonparticipants in 1200-IU D_3_ group (*p* < 0.05).

### Biochemical analysis

Serum 25(OH)D concentrations of all the serum samples were analyzed at the Pediatric Research Centre, University of Helsinki, using a fully automated IDS-iSYS immunoassay system with chemiluminescence detection (Immunodiagnostics System). Further description of biochemical analyses is given in Online Resource Appendix 2, and elsewhere [24].

### Outcome measures

#### Performance-based intelligence quotient and executive functioning

Performance-based neurocognitive abilities were assessed with WISC-IV [Wechsler Intelligence Scale for Children] [25] and NEPSY-II [A Developmental Neuropsychological Assessment–Second Edition] subtests [26]. WISC-IV is a widely used assessment tool for children’s intellectual ability and validated in Finnish population with Finnish norms [25]. We used a short-form [27] including Total Intelligence quotient (IQ) and four indices. Indices were (1) Verbal Comprehension Index (VCI) including Similarities and Vocabulary subscales, (2) Perceptual Reasoning Index (PRI) including Block design and Matrix reasoning subscales, (3) Working Memory Index (WMI) including Digit Span subscale, and (4) Processing Speed Index (PSI) including Coding and Symbol search). Age-standardized scores were used.

NEPSY-II is a neuropsychological test battery for children aged 3–16 years validated in Finnish population with Finnish norms [26]. We assessed four subtests: Design Fluency, Inhibition (Inhibition Naming, Inhibition Inhibition, and Inhibition Switching), Word Generation, and Memory for Faces. Age-standardized scores were used. Exploratory factor analysis was conducted for NEPSY-II subtests to lower the number of variables to avoid multiple testing. One factor accounting for 19% of the total variance was retained (Online Resource: Appendix 3), and it was named performance-based executive functions. Factor score was used for further analysis.

#### Parent-rated executive functioning

Parent-rated executive functioning was assessed with Behavior Rating Inventory of Executive Function (BRIEF) questionnaire, a well-validated questionnaire for children from 5 to 18 years assessing executive functions in everyday life [28]. The scale comprises three indices: the Behavioral Regulation Index (BRI), Metacognition Index (MI) and an overall Global Executive Composite (GEC) [28]. Raw scores of BRIEF were used in all analyses because standardized norms in Finnish samples are unavailable. Higher scores reflect more challenges with executive functioning. The internal consistency of BRIEF was good (Cronbach’s α for GEC = 0.96, MI = 0.94, and BRI = 0.93).

### Statistical analysis

Characteristics between two intervention groups were compared using 2-tailed independent samples t-tests and Pearson χ2-tests. Differences between intervention groups in three primary outcomes of interest, i.e. Total IQ score, Performance-based executive functioning factor score and Parent-rated GEC score, were tested with linear regression analysis.

Linear regression analyses were also used to study the association between prenatal maternal 25(OH)D concentration and child’s 25(OH)D concentration at 12 and 24 months of age and three neurocognitive outcome variables in separate models. We also tested a quadratic association by adding a quadratic term of 25(OH)D into the regression model as some evidence suggests that both low and high 25(OH)D concentrations could be associated with poorer neurodevelopmental outcomes [29, 30]. We used centered variables in testing quadratic associations to avoid multicollinearity between non-quadratic 25(OH)D and quadratic 25(OH)D terms. To assess if the consequent improvement in model fit would be due to overfitting, we performed leave-one-out-cross-validation (LOOCV) and calculated root mean squared error (RMSE), mean absolute error (MAE) and R². To disentangle independent effects of prenatal maternal 25(OH)D concentration and child’s 25(OH)D concentration, we simultaneously added both maternal 25(OH)D concentration and child’s concentration at 12 and 24 months, respectively, in the same models. In addition, we tested for interaction between vitamin D and child’s sex in the primary models. As post-hoc sensitivity analyses, we identified and truncated outliers with the variables with significant associations. The rationale for sensitivity analysis is presented in Online Resource Appendix 4.

As secondary outcomes, we used indices of WISC-IV and BRIEF, running the same analyses as with the primary outcomes.

Associations are presented as crude models (Model I) and after adjustments for child’s sex, parental education, mother’s BMI, and season of birth (Model II). Please see Online Resource Appendix 5 for details on covariate selection and data. For interpretability, we present the change in outcome variable scores per 10 nmol/l increase in 25(OH)D concentration. Missing values were not allowed in the analyses of performance-based neurocognitive abilities (WISC-IV, NEPSY-II). In analyses of parent-rated executive functioning, missing values were imputed according to BRIEF manual instructions [28] if the participant had less than 14 non-response items (< 20%). Imputation was done for 80 participants.

SPSS (IBM SPSS Statistics for Windows, version 28) and R version 4.3.0 (R Core Team, 2023) were used in statistical analyses.

## Results

### Characteristics

Table 1 presents the baseline characteristics of the 398 children according to the intervention groups. The baseline characteristics did not vary by the intervention groups (p-values>0.55).

**Table 1:** Baseline characteristics

	**400-IU group** (*n* = 189) **n(%)/mean(SD)**	**n**	**1200-IU group **(*n* = 209) **n(%)/mean(SD)**	**n**	**p** ^*a*^
**Child**					
Female sex	90 (47.6)	189	100 (47.8)	209	0.96
Gestational age, days	280.5 (7.7)	189	281.4 (7.2)	209	0.23
Parity^b^	1.4 (0.6)	188	1.5 (0.7)	209	0.17
Season of birth		189		209	0.55
Winter	30 (15.9)		40 (19.1)		
Spring	75 (39.7)		70 (33.5)		
Summer	46 (24.3)		58 (27.8)		
Autumn	38 (20.1)		41 (19.6)		
**Mother**					
Age	31.64 (4.0)	189	31.44 (4.4)	209	0.63
Non-smoking	166 (88.8)	187	182 (88.8)	205	> 0.99
BMI	23.3 (3.7)	189	23.4 (3.7)	209	0.82
25(OH)D	83.6 (23.6)	161	83.9 (19.7)	173	0.90
<50 nmol/L	7 (4.3)		7 (4.0)		
50–75 nmol/L	54 (33.5)		47 (27.2)		
>75 nmol/L	101 (62.7)		119 (68.8)		

^a^Group differences calculated with t-test or χ2 -test

SD = Standard Deviation, BMI = body mass index, 25(OH)D = 25-hydroxyvitamin D, ^b^number of siblings

Table 2 presents the follow-up characteristics of the children according to the intervention groups. Children’s 25(OH)D concentration was higher in the 1200-IU than in the 400-IU D_3_ supplementation group at 12 months and at 24 months (p-values < 0.001). Deficiency was rare in both groups (400-IU: 1.6–2.3%, 1200-IU: 0.0%). Performance-based neurocognitive abilities were measured at mean age of 7.7 years (SD = 0.3; range = 7.0–8.9.0.9) and parent-rated questionnaire was collected at mean age of 7.2 years (SD = 0.4; range = 6.3–8.1). Total IQ scores ranged from 73 to 130 and Performance-based executive functions (NEPSY-II) scores ranged from 38 to 88 indicating no extremely low scores [25, 26]. Parent-rated GEC scores ranged from 74 to 172 at the 6–8 years-of-age follow-up

**Table 2:** Follow-up characteristics

	**400-IU group** (*n* = 189) **n(%)/mean(SD)**		**n**	**1200-IU group** (*n* = 209) **n(%)/mean(SD)**	**n**	**p** ^*a*^
**12-month follow-up**						
25(OH)D	84.9 (20.6)		176	117.9 (28.7)	196	**< 0.001**
<50 nmol/L	4 (2.3)			0 (0.0)		
50–75 nmol/L	59 (33.5)			6 (3.1)		
>75 nmol/L	113 (64.2)			190 (96.9)		
**24-month follow-up**						
25(OH)D	87.7 (20.0)		188	121.5 (25.5)	207	**< 0.001**
<50 nmol/L	3 (1.6)			0 (0.0)		
50–75 nmol/L	50 (26.6)			6 (2.9)		
>75 nmol/L	135 (71.8)			201 (97.1)		
Breastfed, months	11.3 (5.6)		188	11.2 (5.5)	209	0.89
Parent’s education level, high^b^	163 (86.7)		188	182 (87.1)	209	0.91
**6–8 years-of-age follow-up**						
Age at neurocognitive testing	7.7 (0.3)		134	7.7 (0.3)	159	0.85
Age at completing BRIEF	7.2 (0.4)		159	7.1 (0.4)	165	0.90
Total IQ score (WISC-IV)	101.4 (12.5)		127	102.5 (11.5)	151	0.43
Performance-based executive functions (NEPSY-II) score	64.3 (9.1)		94	65.1 (8.9)	116	0.49
Parent-rated executive functions (BRIEF, GEC score)	114.9 (21.0)		156	114.7 (21.8)	164	0.86

^a^Group differences calculated with t-test or χ2 -test, ^b^University degree

SD = Standard Deviation, 25(OH)D = 25-hydroxyvitamin D, GEC = Global Executive Composite

### Vitamin D supplementation during early childhood and neurocognitive abilities at ages 6–8 years

**Fig. 2:**
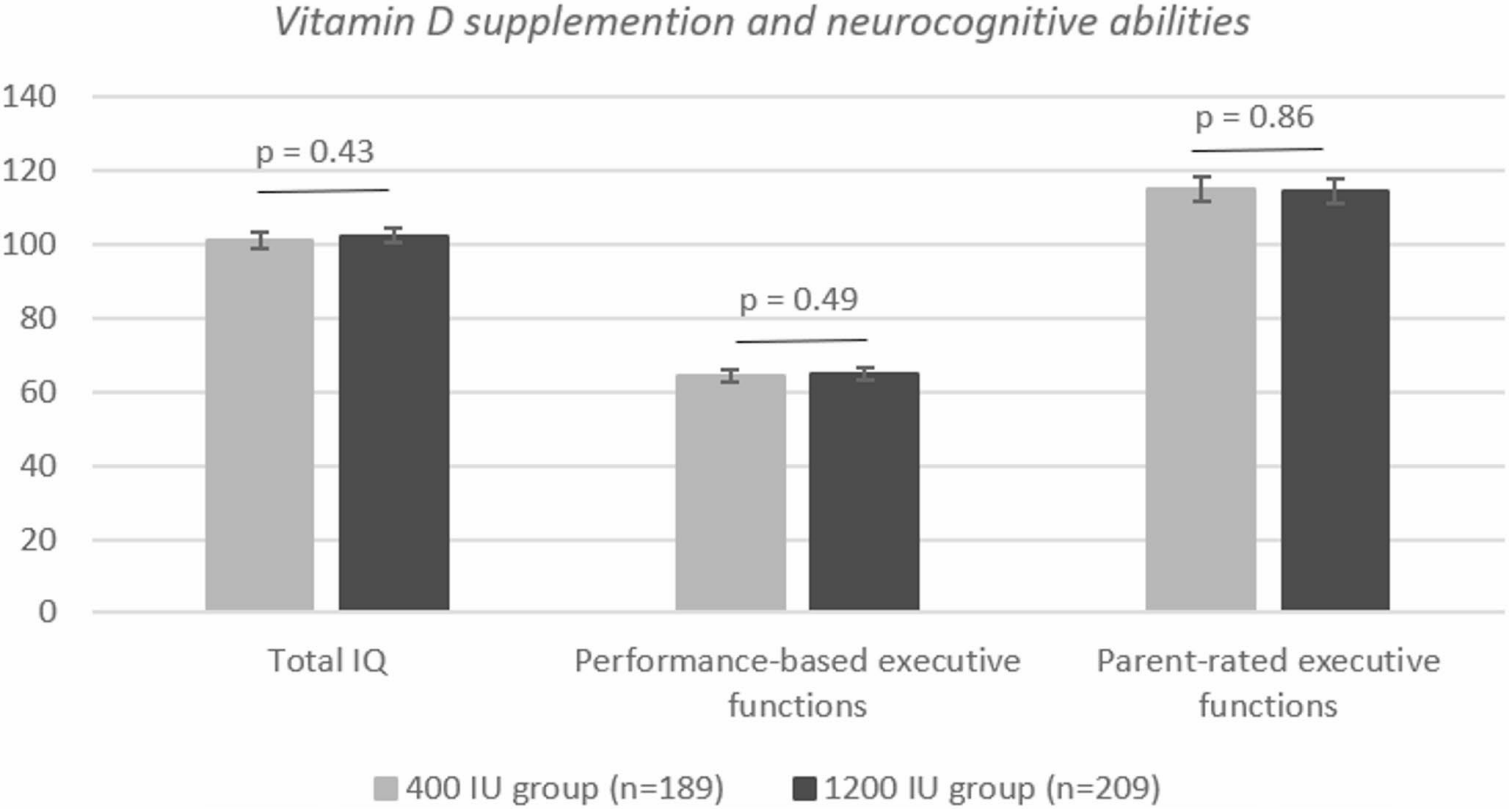
Means of neurocognitive abilities at 6–8 years of age and their 95% confidence intervals by vitamin D supplementation groups

Intervention groups did not differ in Total IQ (β = 1.14, 95% CI [−1.69; 3.97], *p* = 0.43), in performance-based executive functions factor score (β =−0.07, 95% CI [−0.14; 0.28], *p* = 0.49), or in parent-rated GEC score (β =−0.42, 95% CI [−5.13; 4.28], *p* = 0.86) (Fig. 2). Adjusting for covariates did not change the results (p-values > 0.42) and the results did not vary by child’s sex (Online Resource Table 3). Further, there were no group differences in indices of WISC-IV or BRIEF (all p-values > 0.31 in crude and adjusted models, Online Resource Table 4).

### Maternal and child’s early childhood 25(OH)D concentration and Total IQ scores at 6–8 years of age

**Fig. 3:**
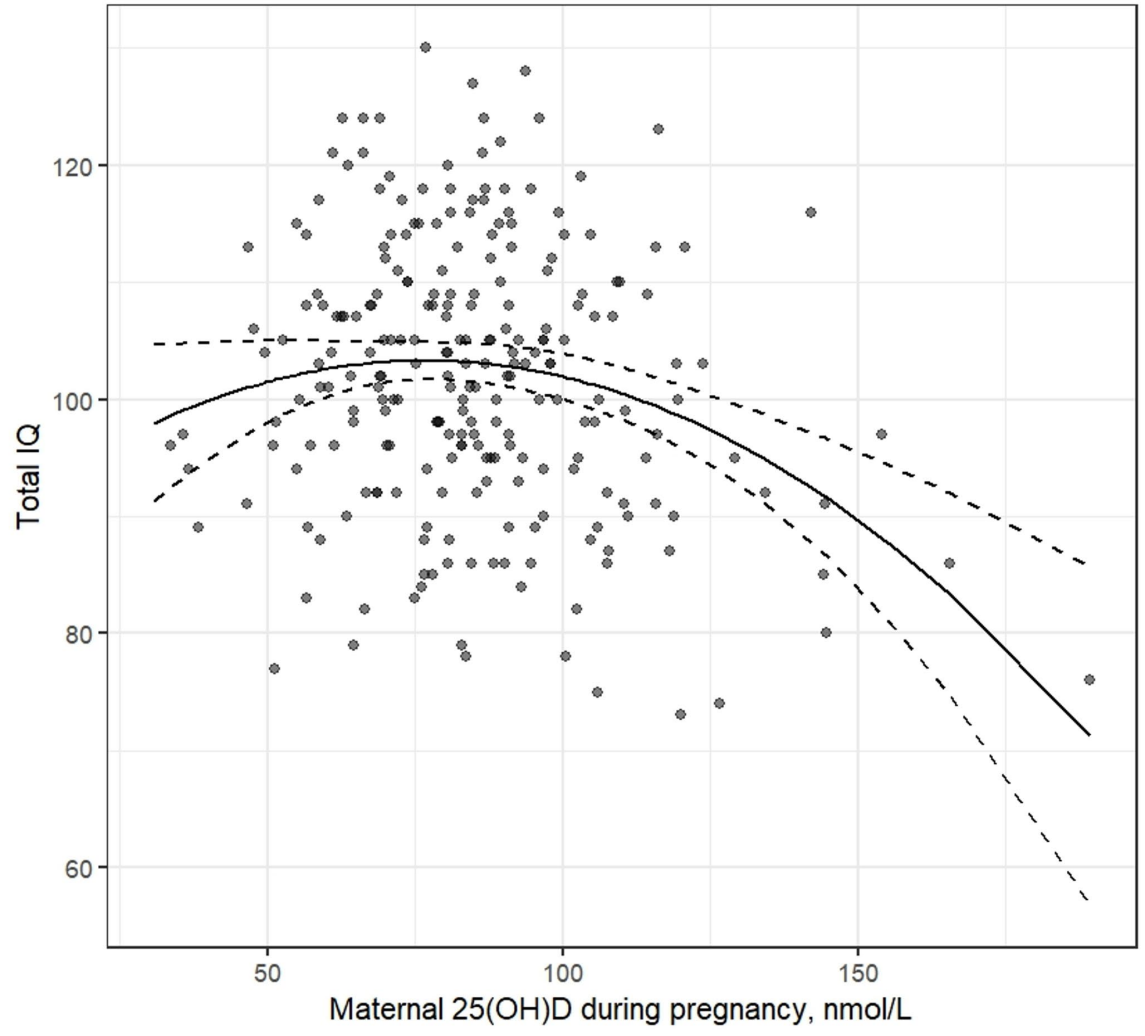
Association between maternal 25(OH)D concentration during pregnancy. Results showed that the vertex of the curve was at 76.64 nmol/L (95% CI:59.64; 93.64 nmol/L)

Table 3 shows that higher prenatal maternal 25(OH)D concentration was associated with the child’s lower Total IQ score at the age of 6–8 years. However, also the quadratic term of maternal 25(OH)D concentration during pregnancy was significantly associated with Total IQ scores (Table 3). The model with the quadratic association showed superior fit over the model with only linear association, without indication that this would be due to overfitting (linear association: RMSE = 11.59, R²=0.02, MAE = 9.41, quadratic association: RMSE = 11.36, R²=0.06, MAE = 9.16). The association visualized in Fig. 3 shows that both lower and higher concentrations were associated with lower Total IQ scores, the vertex of the curve being at 76.64 nmol/L (95% CI: 59.64; 93.64 nmol/L). Mean of Total IQ score was 103.61 (SD = 11.15) in children with mothers in the 95% CI of the vertex, and 99.65 (SD = 10.18) and 98.99 (SD = 12.76) in children above and below the 95% CI of the vertex, respectively. The interpretation of the results did not change when child’s 25(OH)D concentration at 12 or at 24 months was included in the model (Online Resource Table 5, and 6) or with the sensitivity analysis in which outliers (25(OH)D > 150 nmol/L, *n* = 3) were truncated (Model 1, p-value of the quadratic term = 0.003, see all results and figure in Online Resource Appendix 6).

There were no linear or quadratic associations between child’s 25(OH)D concentration at 12 or 24 months and Total IQ scores (all Model 2 p-values > 0.24, Table 2a and Online Resource Table 5, and 6). No interactions between 25(OH)D and sex were found (Online Resource Table 3).

Associations between maternal prenatal and child’s 12- and 24-months’ 25(OH)D concentrations and WISC-IV indices VCI, PRI, WMI, and PSI scores respectively are shown in Online Resource Table 7. Similarly to Total IQ, there were significant quadratic associations between prenatal 25(OH)D and VCI, PRI, and WMI, respectively (all Model 2 p-values < 0.046). However, no association was found between prenatal 25(OH)D and PSI (Model 2 *p* = 0.28). There were no associations between child’s 25(OH)D concentration at 12 or 24 months and VCI, PRI, WMI, and PSI scores (all Model 2 p-values < 0.22).

**Table 3:** Associations between 25(OH)D concentration and total IQ scores. Change in scores per 10 nmol/l increase in 25(OH)D concentration

	Model 1			Model 2	
	β(95% CI)	*p*		β(95% CI)	*p*
Linear associations					
Maternal 25(OH)D during pregnancy	-0.93 (-1.59; -0.27)	**0.006**		-1.03 (-1.70; -0.37)	**0.003**
Child’s 25(OH)D,12 months	-0.19 (-0.66; 0.27)	0.42		-0.21 (-0.67; 0.26)	0.39
Child’s 25(OH)D,24 months	0.10 (-0.38; 0.58)	0.67		-0.06 (-0.54; 0.43)	0.82
***Quadratic associations***					
Maternal 25(OH)D during pregnancy	-0.03 (-0.04; -0.01)	**0.001**		-0.03 (-0.04; -0.01)	**< 0.001**
Child’s 25(OH)D,12 months	0.01 (-0.00; 0.01)	0.30		0.01 (-0.00; 0.02)	0.24
Child’s 25(OH)D,24 months	0.00 (-0.01; 0.02)	0.75		0.00 (-0.01; 0.02)	0.56

Model 1: crude, Model 2: Child’s sex, parent’s education, mother’s BMI, and season of birth controlled

Quadratic associations: Quadratic term added to the linear models, β: non-standardized, CI: Confidence Interval, 25(OH)D = 25-hydroxyvitamin D

### Maternal and early childhood 25(OH)D concentration and executive functioning at ages 6–8 years

There was a linear association between child’s higher 25(OH)D concentration at 12 months and lower GEC score indicating less challenges in executive functioning (β=−0.84 95% CI:−1.67;−0.02, *p* = 0.045). The association attenuated after controlling for covariates (*p* = 0.083). No other linear or quadratic associations were found between maternal 25(OH)D concentration during pregnancy or child’s 12 or 24 months 25(OH)D concentration and executive functioning (Table 4, all Model 1 p-values > 0.06). When modelled simultaneously with maternal 25(OH)D concentration during pregnancy, child’s 25(OH)D at 12 months was associated with performance-based executive functioning factor score before (*p* = 0.028) but not after controlling for covariates (*p* = 0.15). No other associations were found in simultaneous modelling (For all associations, Online Resource Table 5, and 6). No associations between 25(OH)D concentrations and GEC subscales (Online Resource Table 8, all Model 1 p-values > 0.05) and no interactions between 25(OH)D and sex were found (Online Resource Table 3).

**Table 4:** Associations between 25(OH)D concentration and executive functions scores. Change in scores per 10 nmol/l increase in 25(OH)D concentration

	Model 1				Model 2			
	β(95% CI)	*p*			β(95% CI)			*p*
Performance-based executive functions								
***Linear associations***								
Maternal 25(OH)D during pregnancy	−0.05 (−0.10; 0.00)	0.06			−0.03 (−0.08; 0.02)			0.25
Child’s 25(OH)D,12 months	0.01 (−0.03; 0.04)	0.76			0.00 (−0.03; 0.04)			0.85
Child’s 25(OH)D,24 months	0.01 (−0.02; 0.05)	0.52			−0.00 (−0.04; 0.03)			0.86
***Quadratic associations***								
Maternal 25(OH)D during pregnancy	−0.00 (−0.00; 0.00)	0.10			−0.00 (−0.00; 0.00)			0.07
Child’s 25(OH)D,12 months	0.00 (−0.00; 0.00)	0.23			0.00 (−0.00; 0.00)			0.27
Child’s 25(OH)D,24 months	−0.00 (−0.00; 0.00)	0.30			−0.00 (−0.00; 0.00)			0.22
***Parent-rated executive functions (GEC score)***								
***Linear associations***								
Maternal 25(OH)D during pregnancy	−0.65 (−1.81; 0.52)	0.27			−0.61 (−1.74: 0.52)			0.29
Child’s 25(OH)D,12 months	−0.84 (−1.67; −0.02)	**0.045**			−0.69 (−1.47; 0.09)			0.083
Child’s 25(OH)D,24 months	−0.30 (−0.12; 0.06)	0.49			−0.42 (−1.24; 0.40)			0.32
***Quadratic associations***								
Maternal 25(OH)D during pregnancy	0.02 (−0.01; 0.04)	0.22			0.01 (−0.02; 0.03)			0.63
Child’s 25(OH)D,12 months	−0.00 (−0.02; 0.02)	0.79			−0.00 (−0.01; 0.02)			0.78
Child’s 25(OH)D,24 months	−0.01 (−0.02; 0.03)	0.50			0.01 (−0.02; 0.03)			0.65

Model 1: crude, Model 2: Child’s sex, mother’s education, mother’s BMI, and season of birth controlled

Quadratic associations: Quadratic term added to the linear models, 25(OH)D = 25-hydroxyvitamin D

Performance-based executive functions: factor including NEPSY-II subtests (Design Fluency, Inhibition, Word Generation, and Memory for Faces), GEC = Global Executive Composite, β: non-standardized

## Discussion

We studied the impact of three times higher than recommended vitamin D_3_ supplementation between two weeks and two years on neurocognitive abilities, i.e., general cognitive abilities and executive functioning, at 6–8 years of age. We did not find differences on any neurocognitive abilities between vitamin D supplementation groups (1200-IU/day vs. 400-IU/day). 25(OH)D concentration measurements did not associate with neurocognitive abilities after adjusting for covariates, with one exception. We found a quadratic association between maternal 25(OH)D concentration during pregnancy and child’s total IQ score indicating that both lower and higher prenatal maternal 25(OH)D concentrations were associated with lower IQ scores in middle childhood. However, the difference in IQ scores is small, with a mean difference of approximately 4 points, when comparing children whose mothers had medium 25(OH)D concentrations during pregnancy (95% CI: 60–94 nmol/L, vertex at 77 nmol/L) to those with lower or higher concentrations.

Our results are in line with our previous findings from the same vitamin D intervention study that found no beneficial effects of high vitamin D supplementation on parent-reported developmental milestones at the age of one or two [22]. The current results add to the previous findings reporting the non-significant associations using standardized performance-based tests at the early school-age when the environmental demands for academic skills increase. However, there are randomized trial studies that have reported beneficial effects of vitamin D supplementation intervention on neurocognitive abilities [15–18]. Yet, the earlier randomized trials are not readily comparable to the current study as vitamin D supplementation was given during later developmental periods [15–18]. Further, most of the earlier randomized trials do not focus on typically developing children [15, 18] and the ones which do, have relatively small sample sizes (*n* < 120) [16, 17].

The found quadratic association between maternal 25(OH)D concentration during pregnancy and child’s total IQ score aligns with one earlier study showing similar association when neonatal 25(OH)D concentration was used as a proxy of fetal vitamin D status [30]. In addition to that, we are not aware of other studies testing the quadratic association in childhood. There are studies reporting positive association [8–11, 31], no association [32–35], and negative association [36] between maternal 25(OH)D during pregnancy and child’s neurocognitive abilities. Most studies reporting positive association between maternal 25(OH)D concentration and child’s cognitive abilities have focused on vitamin D deficiency and hence used the categorization of 25(OH)D (typical cutoff 50 nmol/L) [11, 30, 31, 37]. There are few studies reporting positive linear association with continuous maternal 25(OH)D concentration during pregnancy and neurocognitive abilities [10, 38, 39]. However, in the studies reporting linear association, 38.0% and 19.5% of the participants had vitamin D insufficiency (< 50 nmol/L) [10, 38], and 25.0% (>80 nmol/L) [10] and 48.9% (>75 nmol/L) [38] were determined vitamin D sufficient contrary to our study of which only 4.2% were insufficient (< 50 nmol/L) and 65.9% had values above 75 nmol/L. In one study reporting positive linear associations, prevalence of vitamin D deficiency was not reported, but median 25(OH)D concentration was within vitamin D deficiency range (45 nmol/L vs. 83.7 nmol/L in our sample) [39]. Finnish vitamin D deficiency levels are generally low in global comparison [40]. It is possible that association between maternal 25(OH)D concentration during pregnancy and child’s cognitive abilities is positive with samples with high rates of vitamin D deficiency and quadratic association becomes visible in samples with more vitamin D sufficient mothers included. Indeed, positive association is found especially in large, diverse populations [41–43]. Maternal skin color does not appear to modify the association, but lower vitamin D deficiency rates in white populations may obscure it [42, 43]. However, there are also other explanations for the discrepancy of the study literature, such as differences in the assessment methods of cognitive abilities and measurement points of 25(OH)D during pregnancy.

Our non-significant findings between 25(OH)D concentration during early childhood and later neurocognitive abilities are in line with some studies [12, 13], but many studies report a positive association [15–17, 44, 45]. In our sample, only 1.1% of children at 12 months and 0.8% of children at 24 months had vitamin D deficiency, which is due to all children being randomized to take either 400-IU or 1200-IU vitamin D supplementation. It is possible that only clear deficiency of vitamin D negatively impacts the development of neurocognitive abilities and that there are no additional benefits above certain 25(OH)D concentrations.

### Strengths and limitations

There are several strengths in our study, including the double-blind randomized clinical trial design, a long follow-up, well-characterized sample, and use of standardized and validated performance-based tests and parent-rated questionnaires [24]. However, there are also limitations, such as attrition, which might influence the generalizability of the results. Of the original sample (*n* = 987) 55.3% (*n* = 546) remained in the follow-up study, and 74% of those families (*n* = 404) were included in the current study. However, the drop-out rate was similar between the supplementation groups and baseline characteristics for nonparticipants did not differ between supplementation groups, suggesting that the attrition did not affect the supplementation groups differently and hence, the results of the intervention.

The sample had a very low percentage of vitamin D deficiency in every measurement point, which might have limited our analysis. However, in the current study we were able to test associations with neurocognitive abilities in a sample with more optimal levels according to current vitamin D concentration recommendations set forth by bone health [46]. In addition, the generalizability of the results might be affected by the high ethnic homogeneity in our sample and national food fortification and promotion of vitamin D supplementation for all as part of the public health efforts in Finland [47, 48]. Future studies should examine whether similar findings emerge in other settings and with confounding factors, such as recommended sunscreen use [49], skin tone, neighborhood or school environment and home cognitive stimulation, which we were not able to test.

## Conclusions

In this secondary analysis of randomized clinical trial, we found no effect of early childhood vitamin D supplementation on neurocognitive abilities at age 6–8 years. However, both low and high prenatal maternal vitamin D levels during pregnancy were associated with lower IQ scores supporting the hypothesis of the role of vitamin D during fetal development. More long-lasting vitamin D randomized trial studies targeting child populations across geographical latitudes and varying food fortification practices are still needed. Future research should explore the optimal 25(OH)D concentrations during pregnancy and early childhood to add knowledge of the multifaceted effects of vitamin D and to inform vitamin D supplementation recommendations.

## Supplementary Information

Below is the link to the electronic supplementary material.

Supplementary Material 1

## Data Availability

The data and codes supporting the ﬁndings are available on request from the corresponding author. The data are not publicly available due to privacy restrictions. Data requests may be subject to review by the Finnish national register authorities and ethical committees.
