# Supplementary material for: Early life vitamin D and neurocognitive abilities at age 6–8 years: a randomized clinical trial and observational analysis
Source: Eur Child Adolesc Psychiatry. 2025 Oct 10;35(2):587–97. doi: 10.1007/s00787-025-02891-7 (PMC12957056; doi:10.1007/s00787-025-02891-7)
Supplement: Supplementary file 1 — Supplementary Material 1 [file 787_2025_2891_MOESM1_ESM.docx]

Journal: European Child & Adolescent Psychiatry

**Early life vitamin D and neurocognitive abilities at the age of 6-8 years: Randomized clinical trial and observational analysis**

Vilja Seppälä, Samuel Sandboge, Elisa Holmlund-Suila, Helena Hauta-alus, Sakari Lintula, Eero Kajantie, Outi Mäkitie, Sture Andersson, Katri Räikkönen, Kati Heinonen

**Correspondence to:**

Vilja Seppälä

[vilja.seppala@tuni.fi](mailto:vilja.seppala@tuni.fi)

Welfare Sciences, Faculty of Social Sciences,

Tampere University, Finland

**Supplementary Material**

Contents of Supplementary Material

Table 1…………………………………………..1

Table 2…………………………………………..2

Appendix 1……………………………………...2

Appendix 2……………………………………...3

Appendix 3……………………………………...3

Appendix 4.……………………………………..5

Appendix 5……………………………………...5

Table 3………………………………………....10

Table 4..………………………………………..11

Table 5..………………………………………..11

Table 6..………………………………………..12

Appendix 6…………………………………….13

Table 7..………………………………………..14

Table 8..………………………………………..15

| **Table 1** Comparison of study participants against cohort members who could not be included due to missing data (attrition group) | | | | | | |
| --- | --- | --- | --- | --- | --- | --- |
|  | **Analytic sample (n=398)** |  | **Attrition sample**  **(n=579)** |  | **Group difference** |  |
|  | **n (%) / mean (SD)** | **n** | **n (%) / mean (SD)** | **n** | **p^a^** |  |
| Sex, female | 190 (47.7) | 398 | 295 (50.9) | 579 | 0.32 |  |
| Gestational age, days | 281.0 (7.5) | 398 | 281.5 (7.8) | 576 | 0.31 |  |
| Parity^b^ | 1.4 (0.8) | 397 | 1.6 (0.7) | 494 | **0.008** |  |
| Season of birth |  | 398 |  | 578 | **0.009** |  |
| winter | 70 (17.6) |  | 119 (20.6) |  |  |  |
| spring | 145 (36.4) |  | 256 (44.3) |  |  |  |
| summer | 104 (26.1) |  | 114 (19.7) |  |  |  |
| autumn | 79 (19.8) |  | 89 (15.4) |  |  |  |
| Parent’s education level, high^c^ | 345 (86.9) | 397 | 369 (76.2) | 484 | **<0.001** |  |
| Mother’s age, years | 31.5 (4.2) | 401 | 30.9 (4.6) | 478 | **0.026** |  |
| Mother’s smoking, No | 348 (88.8) | 392 | 392 (82.0) | 478 | **0.005** |  |
| Breastfeeding, months | 11.3 (5.5) | 397 | 10.1 (5.6) | 457 | **0.003** |  |
| Mother’s BMI | 23.4 (3.7) | 398 | 23.1 (3.7) | 492 | 0.39 |  |
| Maternal 25(OH)D during pregnancy, nmol/L | 83.8 (21.7) | 334 | 80.5 (19.0) | 475 | **0.025** |  |
| < 50 nmol/L | 14 (4.2) |  | 14 (2.9) |  |  |  |
| 50-75 nmol/L | 100 (29.9) |  | 176 (37.1) |  |  |  |
| > 75 nmol/L | 220 (65.9) |  | 285 (60.0) |  |  |  |
| 25(OH)D at 12 mo follow-up, nmol/L | 102.3 (30.1) | 372 | 96.0 (27.8) | 432 | **0.003** |  |
| < 50 nmol/L | 4 (1.1) |  | 6 (1.4) |  |  |  |
| 50-75 nmol/L | 65 (17.5) |  | 98 (22.7) |  |  |  |
| > 75 nmol/L | 303 (81.5) |  | 328 (75.9) |  |  |  |
| 25(OH)D at 24 mo follow-up, nmol/L | 105.5 (28.6) | 395 | 99.3 (26.9) | 419 | **0.002** |  |
| < 50 nmol/L | 3 (0.8) |  | 2 (0.5) |  |  |  |
| 50-75 nmol/L | 56 (14.2) |  | 77 (18.4) |  |  |  |
| > 75 nmol/L | 336 (85.1) |  | 337 (80.4) |  |  |  |
| Total IQ score, 6-8 age follow-up | 102.0 (11.9) | 278 | - | 0 |  |  |
| Tested executive functions (NEPSY-II) score, 6-8 age follow-up | 64.7 (8.96) | 210 | - | 0 |  |  |
| Parent-rated executive functions (BRIEF-P) score, 6-8 age follow-up | 112.72 (21.4) | 320 | - | 0 |  |  |

^a^Group differences calculated with t-test or χ2 -test, ^b^how many siblings at birth, ^c^University degree, SD=Standard Deviation, BMI=body mass index, 25(OH)D=25-hydroxyvitamin D

| **Table 2** Baseline characteristics of attrition group by Vitamin D supplementation groups | | | | | | |
| --- | --- | --- | --- | --- | --- | --- |
|  | **400 IU group (n=237)**  **n (%) / mean (SD)** | **n** | **1200 IU group (n=221)**  **n (%) / mean (SD)** | **n** | **p***^a^* |  |
| Sex, female | 126 (53.2) | 237 | 118 (53.4) | 221 | 0.96 |  |
| Gestational age, days | 280.9 (7.8) | 233 | 282.0 (7.9) | 215 | 0.13 |  |
| Parity^b^ | 1.4 (0.6) | 236 | 1.7 (0.9) | 220 | **0.001** |  |
| Season of birth |  | 237 |  | 221 | 0.24 |  |
| winter | 58 (24.5) |  | 42 (19.0) |  |  |  |
| spring | 98 (41.4) |  | 110 (49.8) |  |  |  |
| summer | 46 (19.4) |  | 35 (15.8) |  |  |  |
| autumn | 35 (14.8) |  | 34 (15.4) |  |  |  |
| Parent’s education level, high^c^ | 157 (77.0) | 204 | 138 (78.0) | 177 | 0.82 |  |
| Mother’s age, years | 30.3 (4.3) | 232 | 31.6 (4.7) | 221 | **0.003** |  |
| Mother’s smoking, No | 191 (83.8) | 228 | 181 (82.6) | 219 | 0.75 |  |
| Breastfeeding, months | 9.9 (5.7) | 230 | 10.5 (5.6) | 216 | 0.21 |  |
| Mother’s BMI | 23.2 (3.7) | 235 | 23.3 (3.7) | 218 | 0.77 |  |
| Maternal 25(OH)D during pregnancy, nmol/L | 81.8 (21.1) | 205 | 80.4 (15.7) | 177 | 0.46 |  |

^a^Group differences calculated with t-test or χ2 -test, ^b^how many siblings at birth, ^c^University degree, SD=Standard Deviation, BMI=body mass index, 25(OH)D=25-hydroxyvitamin D

**Appendix 1** Study protocol: Vitamin D3 supplementation

Infants were randomized on 1:1 basis to receive either 400-IU or 1200-IU of vitamin D_3_ supplementation daily from age 2 weeks to 24 months [1]. Supplementation was administered by the families. Families kept study diary to track treatment compliance, and empty vitamin D bottles were collected at follow-up visits. The vitamin D_3_ dosages were designed to be safe for long-term use and determined based on VIDI pilot study (n=113) described elsewhere [1]. We were unable to add a group with no vitamin D3 supplementation, because it would have been against the Finnish national recommendation on vitamin D supplementation [2] and unethical in northern region where vitamin D from sun light is not sufficient all year around. Parents were asked not to use other vitamin D supplements. No other instructions on nutrition were given. A pharmacist at Helsinki University Hospital, unaffiliated with the study, performed randomization of vitamin D_3_ supplementation in blocks of 50 infants. The vitamin D3 supplements were prepared by Orion Pharmaceuticals and administered once daily (5 drops). Parents received information of the received vitamin D_3_ supplementation after the intervention ended.

**References for Appendix 1**

1. Helve O, Viljakainen H, Holmlund-Suila E et al (2017) Towards evidence-based vitamin D supplementation in infants: vitamin D intervention in infants (VIDI)—study design and methods of a randomised controlled double-blinded intervention study. BMC Pediatr 17:1–8.
2. Jääskeläinen T, Itkonen ST, Lundqvist A et al (2017) The positive impact of general vitamin D food fortification policy on vitamin D status in a representative adult Finnish population: evidence from an 11-y follow-up based on standardized 25-hydroxyvitamin D data. Am J Clin Nutr 105(6):1512–1520. doi:10.3945/ajcn.116.151415.

**Appendix 2** Biochemical analyses

Maternal serum samples were collected as part of routine maternity clinic follow-up visits at 6-27 weeks of gestation (mean = 11.2, standard deviation (SD) = 1.9) and stored in the Finnish Maternity Cohort serum bank, organized by the National Institute for Health and Welfare. Childhood serum samples were collected at ages one and two years. The IDS-iSYS fully automated immunoassay system with chemiluminescence detection (Immunodiagnostic Systems Ltd., Bolton, UK) was used in the analysis of 25-hydroxyvitamin D. The method has been demonstrated to have a good linear agreement with liquid chromatography in tandem with mass spectroscopy (LC-MS, R2=0.942, in-house comparison of 67 samples). Mean (95% Confidence Interval (CI) value for the ratio of IDS-iSYS 25(OH)D to LC-MS 25(OH)D is 0.73 (0.68; 0.78) while intra-assay variations were 7%. Analysis took place at the Pediatric Research Centre, University of Helsinki. Our laboratory participates in the inter-laboratory quality assessment scheme for vitamin D, DEQAS (Charing Cross Hospital, London, UK).

**Appendix 3** Exploratory factor analysis (EFA) for NEPSY-II subtests

Exploratory factor analysis (EFA) was conducted for NEPSY-II [A Developmental Neuropsychological Assessment–Second Edition] subtests to lower the number of variables. EFA was chosen because there were not enough theoretical grounds for using the confirmatory approach to NEPSY-II subtests. Used estimation method was maximum likelihood (ML) with orthogonal rotation (varimax). The best fitting factor solution was chosen after examination of the parallel analysis [1] scree plot, fit indices, eigenvalues of the factors, loadings, communality, uniqueness and complexity of the variables, and interpretability of the factor solution. One factor accounting for 19 % of the total variance was retained (Table 2.1, Table 2.2, Figure 2.1, and Figure 2.2).

**Table 3.1** Exploratory factor analysis (EFA) for NEPSY-II subtests and number of factors. Estimation method was maximum likelihood (ML) with orthogonal rotation (varimax)

| **n of factors** | **χ2 (df)** | **p** | **RMSEA** | **TLI** | **BIC** |
| --- | --- | --- | --- | --- | --- |
| 1 | 15.09 (9) | 0.089 | 0.06 | 0.88 | -34.05 |
| 2 | 6.80 (4) | 0.15 | 0.05 | 0.92 | -16.67 |

RMSEA = residual mean square error of approximation; TLI = Tucker–Lewis index; BIC = Bayesian information criterion


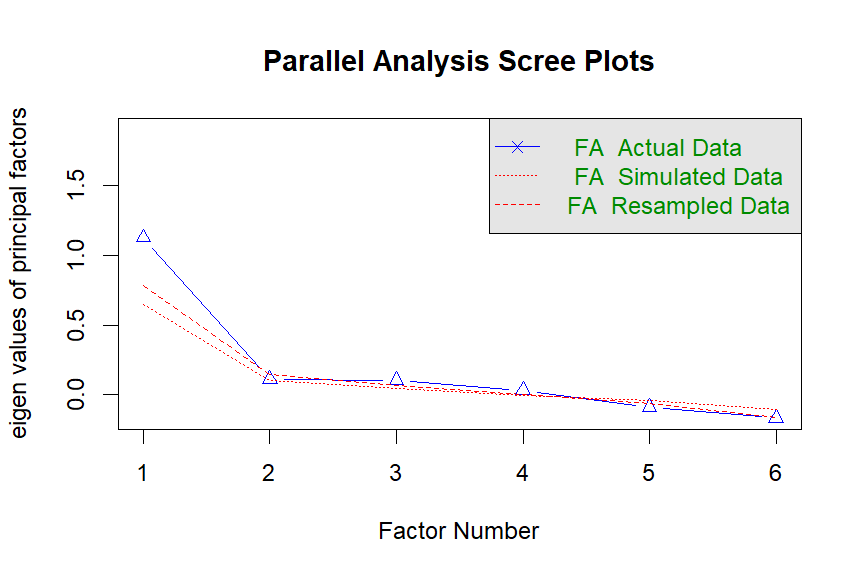


**Figure 3.1** Parallel Analysis Scree Plots of EFA for Neurocognitive subtests (NEPSY-II). Eigenvalue for one factor is greater than 1

**Table 3.2** EFA solution with one factor: Neurocognitive subtests’ (NEPSY-II) and their loadings, communalities, uniqueness values, and complexity values

| **Neurocognitive subtest** | **Factor loading** | **h^2^** | **u^2^** | **com** |
| --- | --- | --- | --- | --- |
| Inhibition Naming | 0.57 | 0.33 | 0.67 | 1 |
| Inhibition Inhibition | 0.50 | 0.25 | 0.75 | 1 |
| Word Generation | 0.46 | 0.21 | 0.79 | 1 |
| Inhibition Switching | 0.45 | 0.20 | 0.80 | 1 |
| Design Fluency | 0.27 | 0.07 | 0.93 | 1 |
| Memory for Faces | 0.25 | 0.06 | 0.94 | 1 |

communalities (h2), error estimates/uniqueness values (u2), complexity values


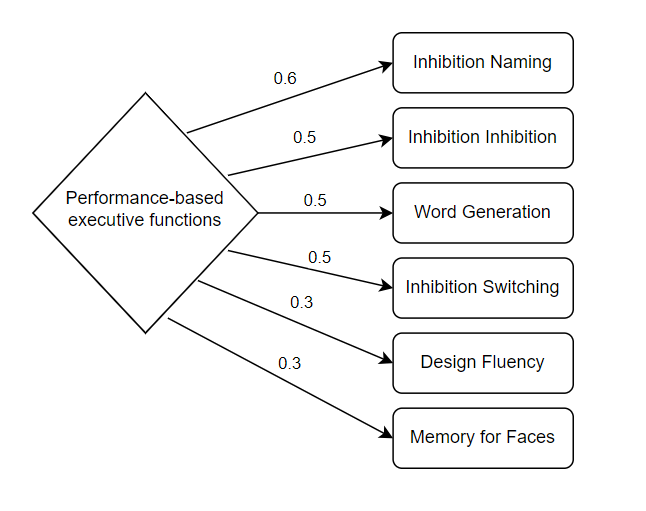


**Figure 3.2** EFA solution with one factor, Performance-based executive functions, and Neurocognitive subtests’ (NEPSY-II) and their loadings

**Reference for Appendix 3**

1. Horn JL (1965) A rationale and test for the number of factors in factor analysis. Psychometrika 30:179–185.

**Appendix 4** Rationale for post-hoc sensitivity analyses between prenatal maternal 25(OH)D concentration and Total IQ at 6-8 years

To assess further the association between prenatal maternal 25(OH)D concentration and Total IQ at 6-8 years of age, which were the only variables of interest with significant associations, we conducted post-hoc sensitivity analyses. We identified and truncated outliers (maternal 25(OH)D concentration values > 150 nmol/L during pregnancy, n=3) using the Mahalanobis method (p<0.01). We truncated outliers for sensitivity analysis, because we acknowledged the excessive influence these outliers could potentially have in the statistical models, even though we had no reason to assume they represented measurement error. We used significance level of p<0.01 for χ2, because for sensitivity analyses the commonly used p<.0.001 might be unnecessary conservative and fail to catch all the outliers with excessive influence on the models.

**Appendix 5** Covariates

We chose 10 potential covariates from VIDI dataset based on subject-matter knowledge. The ones selected were known to be associated with neurocognitive abilities and/or vitamin D levels. They were child’s age, sex [1-2], season of birth [2], gestational age [3], parity [4], mother’s age at delivery [1], smoking [5], body mass index (kg/m2, BMI) [2,6], breastfeeding duration [7], and parental educational level [1]. See Table 5.1 for details on how the covariates were assessed. Then, we tested and chose covariates that were associated with our variables of interest to avoid overfitting of the models and to include the most relevant ones. Child’s sex, parental education, mother’s BMI, and season of birth were associated with one or more variables of interest and chosen for further analyses (See Tables 5.2-5.7 for all the associations). We used the same covariate set systematically in all the analyses to maximize comparability and replicability of the analyses. No missing values were allowed for the covariates. One missing value was observed in parental education level -variable, and thus adjusted models were run excluding the corresponding participant. There were no additional missing values in chosen covariates.

**Table 5.1** Details on on how the covariates were assessed

| **Covariate** | **Assessment** |
| --- | --- |
| Child’s sex | Information at birth, form hospital records |
| Child’s age | Information at assessment time, in years, calculated from assessment dates and from birth date derived from hospital records |
| Child’s season of birth | Season at the time of birth, categorized into winter (Dec, Jan, Feb), spring (Mar, Apr, May), summer (Jun, Jul, Aug) or autumn (Sep, Oct, Nov) |
| Child’s gestational age | Information at birth, in days, from hospital records |
| Parity | Information at birth, number of mothers previous labours (/child’s biological siblings), from hospital records |
| Mother’s age | Information at birth, in years, from hospital records |
| Mother’s smoking | Smoking before pregnancy and/or after the birth of the child, binary variable: yes vs no, self-reported information at birth and if missing, information completed at 24-month follow-up |
| Mother’s body-mass-index | Information from hospital records and if missing, from questionnaires at baseline, body-mass-index (kg/m2) before pregnancy |
| Breastfeeding duration | Mother’s self-reported information from diaries filled up during intervention, in months |
| Parental education level | Information collected from parent’s questionnaires, the latest information known of the highest self-reported education of either or only parent, categorized into no university degree (primary/secondary) vs university degree (lower tertiary/upper tertiary) |

**Table 5.2** Associations between maternal 25(OH)D during pregnancy and covariates

| **M****aternal 25(OH)D during pregnancy** | |  | |  |
| --- | --- | --- | --- | --- |
|  | **β [95% CI]** | | **p** | |
| Sex | -3.69 [-8.36;0.97] | | 0.12 | |
| Parity^a^ | 1.28 [-2.16;4.72] | | 0.47 | |
| Gestational age, days | -0.2 [-0.55;0.07] | | 0.13 | |
| Season of birth |  | | 0.15 | |
| winter | 1.92 [-4.77;8.62] | | 0.57 | |
| spring | ref | | ref | |
| summer | -4.88 [-10.92;1.17] | | 0.11 | |
| autumn | -4.44 [-10.71;1.84] | | 0.17 | |
| Parent’s education level, high/low^b^ | 9.06 [1.84;16.27] | | **0.014** | |
| Mother’s age, years | 0.35 [-0.23;0.93] | | 0.23 | |
| Breastfeeding, months | -0.12 [-0.54;0.30] | | 0.58 | |
| Smoking, yes/no | -0.49 [-8.09;7.10] | | 0.90 | |
| BMI, kg/m2 | -0.72 [-1.33;-0.12] | | **0.020** | |

Linear regression analysis used to test each covariates association with 25(OH)D independently, β: non-standardized, CI=Confidence Interval, 25(OH)D=25-hydroxyvitamin D, BMI=body mass index, ^a^how many siblings at birth, ^b^high=university degree, low=no university degree

**Table 5.3** Associations between 25(OH)D at 12 months and covariates

| **25(OH)D at 12 months** |  |  |
| --- | --- | --- |
|  | **β [95% CI]** | **p** |
| Sex | 2.58 [-3.56; 8.72] | 0.41 |
| Parity^a^ | 2.22 [-2.30;6.74] | 0.34 |
| Gestational age, days | 0.17 [-0.25;0.59] | 0.43 |
| Season of birth |  | 0.24 |
| winter | 8.62 [-0.30;17.54] | 0.058 |
| spring | ref | ref |
| summer | 2.02 [-5.86;9.91] | 0.61 |
| autumn | 5.49 [-2.97;13.95] | 0.20 |
| Parent’s education level, high/low^b^ | -1.41 [-10.55;7.73] | 0.76 |
| Mother’s age, years | 0.22 [-0.52;0.95] | 0.56 |
| Breastfeeding, months | -0.33 [-0.89;0.23] | 0.25 |
| Smoking, yes/no | 0.95 [-8.87;10.76] | 0.85 |
| BMI, kg/m2 | 0.68 [-0.13;1.48] | 0.10 |

Linear regression analysis used to test each covariates association with 25(OH)D independently, β: non-standardized, CI=Confidence Interval, 25(OH)D=25-hydroxyvitamin D, BMI=body mass index, ^a^how many siblings at birth, ^b^high=university degree, low=no university degree

**Table 5.4** Associations between 25(OH)D at 24 months and covariates

| **25(OH)D at 24 months** |  |  |
| --- | --- | --- |
|  | **β [95% CI]** | **p** |
| Sex | 4.81 [-0.84;10.45] | 0.10 |
| Parity^a^ | 1.72 [-2.49;5.93] | 0.42 |
| Gestational age, days | 0.18 [-0.20;0.56] | 0.36 |
| Season of birth |  | **0.003** |
| winter | 5.37 [-2.70;13.44] | 0.19 |
| spring | ref | ref |
| summer | 10.55 [3.43;17.68] | **0.004** |
| autumn | 13.04 [5.22;20.86] | **0.001** |
| Parent’s education level, high/low^b^ | 5.04 [-3.31;13.39] | 0.24 |
| Mother’s age, years | 0.38 [-0.30;1.06] | 0.28 |
| Breastfeeding, months | 0.06 [-0.46;0.57] | 0.82 |
| Smoking, yes/no | -5.07 [-14.02;3.87] | 0.27 |
| BMI, kg/m2 | -0.17 [-0.93;0.59] | 0.66 |

Linear regression analysis used to test each covariates association with 25(OH)D independently, β: non-standardized, CI=Confidence Interval, 25(OH)D=25-hydroxyvitamin D, BMI=body mass index, ^a^how many siblings at birth, ^b^high=university degree, low=no university degree

**Table 5.5** Associations between Total IQ and covariates

| **Total IQ (WISC-IV)** |  |  |
| --- | --- | --- |
|  | **β [95% CI]** | **p** |
| Sex | 2.17 [-0.64;4.98] | 0.13 |
| Parity^a^ | -0.88 [-2.92;1.15] | 0.39 |
| Age, years | 2.23 [-2.30;6.76] | 0.33 |
| Gestational age, days | 0.03 [-0.05; 0.10] | 0.51 |
| Season of birth |  | 0.46 |
| winter | -0.27 [-4.18;3.65] | 0.89 |
| spring | ref | ref |
| summer | 1.73 [-1.88;5.34] | 0.35 |
| autumn | 2.74 [-1.37;6.86] | 0.19 |
| Parent’s education level, high/low^b^ | 7.94 [3.79;12.10] | **<0.001** |
| Mother’s age, years | 0.12 [-0.22;0.46] | 0.49 |
| Breastfeeding, months | 0.27 [0.00;0.53] | 0.050 |
| Smoking, yes/no | -1.79 [-6.34;2.76] | 0.44 |
| BMI, kg/m2 | -0.32 [-0.70;0.06] | 0.10 |

Linear regression analysis used to test each covariates association with Total IQ independently, β: non-standardized, CI=Confidence Interval, BMI=body mass index, ^a^how many siblings at birth, ^b^high=university degree, low=no university degree

**Table 5.6** Associations between Performance-based executive functions (NEPSY-II) and covariates

| **Performance-based executive functions (NEPSY-II)** | |  | |  |
| --- | --- | --- | --- | --- |
|  | **β [95% CI]** | | **p** | |
| Sex | 0.30 [0.09;0.50] | | **0.005** | |
| Parity^a^ | -0.09 [-0.23;0.06] | | 0.23 | |
| Age, years | -0.33 [-0.68;0.02] | | 0.062 | |
| Gestational age, days | 0.44 [-0.94;1.83] | | 0.53 | |
| Season of birth |  | | 0.25 | |
| winter | -0.20 [-0.49;0.10] | | 0.19 | |
| spring | ref | | ref | |
| summer | 0.10 [-0.16;0.35] | | 0.44 | |
| autumn | 0.11 [-0.22;0.43] | | 0.52 | |
| Parent’s education level, high/low^b^ | 0.14 [-0.18;0.46] | | 0.39 | |
| Mother’s age, years | -0.00 [-0.03;0.03] | | 0.96 | |
| Breastfeeding, months | -0.01 [-0.03;0.01] | | 0.44 | |
| Smoking, yes/no | 0.12 [-0.24;0.48] | | 0.50 | |
| BMI, kg/m2 | 0.01 [-0.02; 0.04] | | 0.51 | |

Linear regression analysis used to test each covariates association with Performance-based executive functions independently, β: non-standardized, CI=Confidence Interval, BMI=body mass index, ^a^how many siblings at birth, ^b^high=university degree, low=no university degree

**Table 5.7** Associations between Parent-reported executive functions (BRIEF) and covariates

| **Parent-reported executive functions (BRIEF)** | |  | |  |
| --- | --- | --- | --- | --- |
|  | **β [95% CI]** | | **p** | |
| Sex | -12.93 [-17.43;-8.44] | | **<0.001** | |
| Parity^a^ | -1.98 [-5.57;1.62] | | 0.28 | |
| Age, years | 1.78 [-3.67;7.23] | | 0.52 | |
| Gestational age, days | -0.01 [-0.33;0.31] | | 0.95 | |
| Season of birth |  | | **0.007** | |
| winter | 1.72 [-5.22;8.65] | | 0.63 | |
| spring | ref | | ref | |
| summer | 8.47 [2.51;14.42] | | **0.005** | |
| autumn | 9.10 [2.78;15.41] | | **0.005** | |
| Parent’s education level, high/low^b^ | -1.80 [-8.84;5.24] | | 0.62 | |
| Mother’s age, years | 0.28 [-0.29;0.84] | | 0.34 | |
| Breastfeeding, months | -0.05 [-0.47;0.37] | | 0.82 | |
| Smoking, yes/no | 2.49 [-4.98;9.96] | | 0.51 | |
| BMI, kg/m2 | -0.29 [-0.91;0.32] | | 0.35 | |

Linear regression analysis used to test each covariates association with Parent-reported executive functions independently, β: non-standardized, CI=Confidence Interval, BMI=body mass index, ^a^how many siblings at birth, ^b^high=university degree, low=no university degree

**Reference for Appendix 5**

1. Schoon I, Jones E, Cheng H, Maughan B (2012) Family hardship, family instability, and cognitive development. J Epidemiol Community Health 66(8):716–722.
2. Tolppanen AM, Fraser A, Fraser WD, Lawlor DA (2012) Risk factors for variation in 25-hydroxyvitamin D3 and D2 concentrations and vitamin D deficiency in children. J Clin Endocrinol Metab 97(4):1202–1210.
3. Pierrat V, Marchand-Martin L, Arnaud C et al (2017) Neurodevelopmental outcome at 2 years for preterm children born at 22 to 34 weeks’ gestation in France in 2011: EPIPAGE-2 cohort study. BMJ 358:j3448. doi:10.1136/bmj.j3448.
4. Ning K, Zhao L, Franklin M et al (2020) Parity is associated with cognitive function and brain age in both females and males. Scientific reports 10(1):6100.
5. Polańska K, Jurewicz J, Hanke W (2015) Smoking and alcohol drinking during pregnancy as the risk factors for poor child neurodevelopment—a review of epidemiological studies. Int J Occup Med Environ Health 28(3):419–443. doi:10.13075/ijomeh.1896.00424.
6. Adane AA, Mishra GD, Tooth LR (2016) Maternal pre-pregnancy obesity and childhood physical and cognitive development of children: a systematic review. International journal of obesity 40(11):1608–1618.
7. Horta BL, Loret de Mola C, Victora CG (2015) Breastfeeding and intelligence: a systematic review and meta‐analysis. Acta Paediatr 104:14–19.

**Table 3** Interactions of vitamin D and sex in association to neurocognitive abilities. Change in scores per 10 nmol/l increase in 25(OH)D concentration

|  | **β (independent variable x sex)** | **p** |
| --- | --- | --- |
| **400 IU vs 1200 IU supplementation** |  |  |
| Total IQ | -1.14 | 0.69 |
| Performance-based executive functions (NEPSY-II) | 0.00 | 1.00 |
| Parent-rated executive functions (BRIEF, GEC) | -3.46 | 0.45 |
| **Maternal 25(OH)D during pregnancy** |  |  |
| Total IQ | 1.14 | 0.10 |
| Performance-based executive functions (NEPSY-II) | 0.01 | 0.88 |
| Parent-rated executive functions (BRIEF, GEC) | -0.36 | 0.76 |
| **Child’s 25(OH)D, 12 months** |  |  |
| Total IQ | 0.56 | 0.23 |
| Performance-based executive functions (NEPSY-II) | -0.00 | 0.99 |
| Parent-rated executive functions (BRIEF, GEC) | 1.32 | 0.10 |
| **Child’s 25(OH)D, 24 months** |  |  |
| Total IQ | 0.47 | 0.34 |
| Performance-based executive functions (NEPSY-II) | -0.01 | 0.68 |
| Parent-rated executive functions (BRIEF, GEC) | -0.01 | 0.99 |

β: non-standardized, 25(OH)D=25-hydroxyvitamin D, GEC= Global Executive Composite

| **Table 4** Vitamin D supplementation (400 IU vs 1200 IU) during early childhood and neurocognitive abilities at ages 6-8 years (indices) | | | | | |
| --- | --- | --- | --- | --- | --- |
|  | **Model 1** |  |  | **Model 2** |  |
|  | **β(95% CI)** | **p** |  | **β(95% CI)** | **p** |
|  |  |  |  |  |  |
| **WISC-IV** | | | | | |
| Verbal Comprehension Index | 0.77 (-2.70; 4.24) | 0.66 |  | 0.77 (-2.75; 4.29) | 0.67 |
| Perceptual Reasoning Index | 1.18 (-2.36; 4.72) | 0.51 |  | 1.36 (-2.13; 4.85) | 0.45 |
| Working Memory Index | 1.72 (-1.59; 5.03) | 0.31 |  | 1.62 (-1.68; 4.92) | 0.34 |
| Processing Speed Index | -0.04 (-2.75; 2.67) | 0.98 |  | 0.18 (-2.88; 2.51) | 0.89 |
| **BRIEF** |  |  |  |  |  |
| Behavioral Regulation Index | -0.36 (-2.48; 1.77) | 0.74 |  | -0.34 (-2.36; 1.68) | 0.74 |
| Metacognition Index | 0.04 (-2.92; 3.00) | 0.98 |  | -0.06 (-2.87; 2.75) | 0.97 |

β: non-standardized, CI=Confidence Interval

| **Table 5** Associations between maternal 25(OH)D during pregnancy + child’s 25(OH)D at 12 months (both as independent variables) and total IQ and executive functions. Change in scores per 10 nmol/l increase in 25(OH)D concentration | | | | | |
| --- | --- | --- | --- | --- | --- |
|  | **Model 1** |  |  | **Model 2** |  |
|  | **β(95% CI)** | **p** |  | **β(95% CI)** | **p** |
| ***Linear associations*** |  |  |  |  |  |
| **Total IQ** |  |  |  |  |  |
| Maternal 25(OH)D during pregnancy | -0.94 (-1.61;-0.26) | **0.007** |  | -1.02 (-1.71;-0.33) | **0.004** |
| Child’s 25(OH)D,12 months | -0.04 (-0.52;0.43) | 0.86 |  | -0.04 (-0.52;0.44) | 0.87 |
| **Performance-based executive functions** |  |  |  |  |  |
| Maternal 25(OH)D during pregnancy | -0.06 (-0.11;-0.00) | **0.028** |  | -0.04 (-0.09;0.02) | 0.15 |
| Child’s 25(OH)D,12 months | 0.01 (-0.03;0.04) | 0.59 |  | 0.01 (-0.03;0.04) | 0.67 |
| **Parent-rated executive functions (GEC score)** |  |  |  |  |  |
| Maternal 25(OH)D during pregnancy | -0.67 (-1.87;0.52) | 0.27 |  | -0.61 (-1.78;0.55) | 0.30 |
| Child’s 25(OH)D,12 months | -0.75 (-1.63;0.13) | 0.093 |  | -0.51 (-1.36;0.33) | 0.23 |
| ***Quadratic associations*** |  |  |  |  |  |
| **Total IQ** |  |  |  |  |  |
| Maternal 25(OH)D during pregnancy | -0.03 (-0.04;-0.01) | **<0.001** |  | -0.03 (-0.05;-0.01) | **<0.001** |
| Child’s 25(OH)D,12 months | 0.01 (-0.00;0.01) | 0.27 |  | 0.01 (-0.00;0.02) | 0.20 |
| **Performance-based executive functions** |  |  |  |  |  |
| Maternal 25(OH)D during pregnancy | -0.00 (-0.00;0.00) | 0.11 |  | -0.00 (-0.00;0.00) | 0.084 |
| Child’s 25(OH)D,12 months | 0.00 (-0.00;0.00) | 0.26 |  | 0.00 (-0.00;0.00) | 0.29 |
| **Parent-rated executive functions (GEC score)** |  |  |  |  |  |
| Maternal 25(OH)D during pregnancy | 0.02 (-0.01;0.05) | 0.16 |  | -0.01 (-0.02;0.04) | 0.57 |
| Child’s 25(OH)D,12 months | -0.00 (-0.02;0.02) | 0.73 |  | 0.00 (-0.02;0.04) | 0.90 |

Maternal 25(OH)D during pregnancy and child’s 25(OH)D at 12 months are both included as independent variables in the models. Model 1: crude, Model 2: Child’s sex, parent’s education, mother’s BMI, and season of birth controlled. Quadratic associations: Quadratic term added to the linear models, β: non-standardized, CI: Confidence Interval, 25(OH)D=25-hydroxyvitamin D

| **Table 6** Associations between maternal 25(OH)D during pregnancy + child’s 12(OH)D at 24 months (both as independent variables) and total IQ and executive functions. Change in scores per 10 nmol/l increase in 25(OH)D concentration | | | | | |
| --- | --- | --- | --- | --- | --- |
|  | **Model 1** |  |  | **Model 2** |  |
|  | **β(95% CI)** | **p** |  | **β(95% CI)** | **p** |
| ***Linear associations*** |  |  |  |  |  |
| **Total IQ** |  |  |  |  |  |
| Maternal 25(OH)D during pregnancy | -0.97 (-1.64;-0.30) | **0.005** |  | -1.06 (-1.74;-0.38) | **0.002** |
| Child’s 25(OH)D,24 months | 0.24 (-0.27;0.74) | 0.36 |  | 0.14 (-0.37;0.65) | 0.58 |
| **Performance-based executive functions** |  |  |  |  |  |
| Maternal 25(OH)D during pregnancy | -0.05 (-0.10;-0.00) | 0.050 |  | -0.03 (-0.08;0.02) | 0.25 |
| Child’s 25(OH)D,24 months | 0.02 (-0.02;0.01) | 0.41 |  | 0.00 (-0.03;0.04) | 0.83 |
| **Parent-rated executive functions (GEC score)** |  |  |  |  |  |
| Maternal 25(OH)D during pregnancy | -0.63 (-1.81;0.54) | 0.29 |  | -0.58 (-1.72;0.56) | 0.32 |
| Child’s 25(OH)D,24 months | -0.18 (-1.10;0.74) | 0.70 |  | -0.16 (-1.04;0.73) | 0.73 |
| ***Quadratic associations*** |  |  |  |  |  |
| **Total IQ** |  |  |  |  |  |
| Maternal 25(OH)D during pregnancy | -0.03 (-0.04;-0.01) | **0.002** |  | -0.03 (-0.05;-0.01) | **<0.001** |
| Child’s 25(OH)D,24 months | 0.00 (-0.01;0.02) | 0.84 |  | 0.00 (-0.01;0.02) | 0.61 |
| **Performance-based executive functions** |  |  |  |  |  |
| Maternal 25(OH)D during pregnancy | -0.00 (-0.00;0.00) | 0.13 |  | -0.00 (-0.00;0.00) | 0.11 |
| Child’s 25(OH)D,24 months | -0.00 (-0.00;0.00) | 0.36 |  | -0.00 (-0.00;0.00) | 0.29 |
| **Parent-rated executive functions (GEC score)** |  |  |  |  |  |
| Maternal 25(OH)D during pregnancy | 0.02 (-0.01;0.04) | 0.22 |  | 0.01 (-0.02;0.03) | 0.65 |
| Child’s 25(OH)D,24 months | 0.01 (-0.02;0.03) | 0.69 |  | 0.00 (-0.02;0.03) | 0.82 |

Maternal 25(OH)D during pregnancy and child’s 25(OH)D at 24 months are both included as independent variables in the models. Model 1: crude, Model 2: Child’s sex, parent’s education, mother’s BMI, and season of birth controlled. Quadratic associations: Quadratic term added to the linear models, β: non-standardized, CI: Confidence Interval, 25(OH)D=25-hydroxyvitamin D

**Appendix 6** Results of post-hoc sensitivity analyses between prenatal maternal 25(OH)D concentration during pregnancy and Total IQ at 6-8 years.

Sensitivity analyses did not change the results of the association between prenatal maternal 25(OH)D concentration during pregnancy and Total IQ at 6-8 years (Table 4.1 and Figure 4.1). In the quadratic model, the vertex point of the curve was at 77.01 nmol/L (95 % CI: 65.24; 88.79 nmol/L).

| **Table 6.1** Associations between 25(OH)D concentration and Total IQ scores in sensitivity analysis (maternal 25(OH)D concentration values during pregnancy < 150 nmol/L). Change in scores per 10 nmol/l increase in 25(OH)D concentration | | | | | |
| --- | --- | --- | --- | --- | --- |
|  | **Model 1** |  |  | **Model 2** |  |
|  | **β(95% CI)** | **p** |  | **β(95% CI)** | **p** |
| ***Linear associations*** | | | | | |
| Maternal 25(OH)D during pregnancy | -0.70 (-1.43; 0.04) | **0.063** |  | -0.79 (-1.52; -0.05) | **0.036** |
| ***Quadratic* *associations*** | | | | | |
| Maternal 25(OH)D during pregnancy | -0.04 (-0.06; -0.01) | **0.003** |  | -0.04 (-0.07; -0.02) | **0.002** |

Model 1: crude, Model 2: Child’s sex, parent’s education, mother’s BMI, and season of birth controlled.

Quadratic associations: Quadratic term added to the linear models, β: non-standardized, CI=Confidence Interval, 25(OH)D=25-hydroxyvitamin D


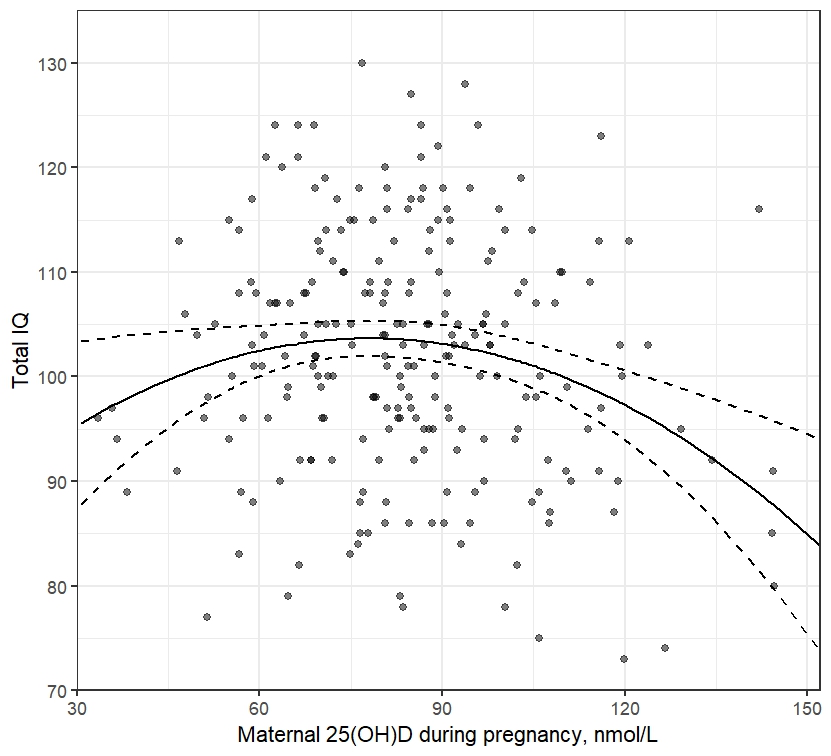


**Figure 6.1** Association between maternal 25(OH)D concentration during pregnancy in sensitivity analysis (maternal 25(OH)D concentration values during pregnancy < 150 nmol/L). Results showed that the vertex of the curve was at 77.01 nmol/L (95 % CI: 65.24; 88.79 nmol/L)

| **Table 7** Associations between 25(OH)D concentration and indices of WISC IV. Change in indices per 10 nmol/l increase in 25(OH)D levels | | | | | |
| --- | --- | --- | --- | --- | --- |
|  | **Model 1** |  |  | **Model 2** |  |
|  | **β(95% CI)** | **p** |  | **β(95% CI)** | **p** |
| **Verbal Comprehension Index** |  |  |  |  |  |
| ***Linear associations*** | | | | | |
| Maternal 25(OH)D during pregnancy | -1.10 (-1.89;-0.30) | **0.007** |  | -1.22 (-2.04;-0.40) | **0.004** |
| Child’s 25(OH)D, 12 months | 0.19 (-0.76; 0.39) | 0.53 |  | -0.20 (-0.79;0.39) | 0.51 |
| Child’s 25(OH)D, 24 months | -0.14 (-0.73;0.45) | 0.64 |  | -0.19 (-0.80;0.43) | 0.55 |
| ***Quadratic* *associations*** | | | | | |
| Maternal 25(OH)D during pregnancy | -0.02 (-0.04;-0.01) | **0.010** |  | -0.03 (-0.05;-0.01) | **0.008** |
| Child’s 25(OH)D, 12 months | 0.01 (-0.00;0.02) | 0.25 |  | 0.01 (-0.01;0.02) | 0.30 |
| Child’s 25(OH)D, 24 months | 0.00 (-0.02;0.02) | 0.84 |  | 0.00 (-0.01;0.02) | 0.79 |
| **Perceptual Reasoning Index** |  |  |  |  |  |
| ***Linear associations*** | | | | | |
| Maternal 25(OH)D during pregnancy | -1.02 (-1.85;-0.18) | **0.017** |  | -1.29 (-2.12;-0.46) | **0.003** |
| Child’s 25(OH)D, 12 months | -0.13 (-0.71;0.45) | 0.66 |  | -0.09 (-0.66;0.49) | 0.77 |
| Child’s 25(OH)D, 24 months | 0.15 (-0.44;0.75) | 0.61 |  | 0.04 (-0.56;0.64) | 0.90 |
| ***Quadratic* *associations*** | | | | | |
| Maternal 25(OH)D during pregnancy | -0.03 (-0.05;-0.01) | **0.006** |  | -0.03 (-0.05;-0.01) | **0.001** |
| Child’s 25(OH)D, 12 months | 0.01 (-0.01;0.02) | 0.36 |  | 0.01 (-0.01;0.02) | 0.32 |
| Child’s 25(OH)D, 24 months | 0.00 (-0.02;0.02) | 0.87 |  | 0.00 (-0.01;0.02) | 0.64 |
| **Working Memory Index** |  |  |  |  |  |
| ***Linear associations*** |  |  |  |  |  |
| Maternal 25(OH)D during pregnancy | -0.01 (-0.82;0.80) | 0.97 |  | -0.11 (-0.93;0.71) | 0.79 |
| Child’s 25(OH)D, 12 months | -0.07 (-0.62;0.49) | 0.82 |  | -0.05 (-0.60;0.51) | 0.87 |
| Child’s 25(OH)D, 24 months | 0.37 (-0.18;0.93) | 0.19 |  | 0.22 (-0.35;0.79) | 0.44 |
| ***Quadratic* *associations*** |  |  |  |  |  |
| Maternal 25(OH)D during pregnancy | -0.02 (-0.04;0.00) | 0.083 |  | -0.02 (-0.04;-0.00) | **0.046** |
| Child’s 25(OH)D, 12 months | -0.00 (-0.01;0.01) | 0.83 |  | 0.00 (-0.01;0.01) | 0.92 |
| Child’s 25(OH)D, 24 months | -0.00 (-0.02;0.01) | 0.81 |  | -0.00 (-0.02;0.02) | 0.93 |
| **Processing Speed Index** |  |  |  |  |  |
| ***Linear associations*** |  |  |  |  |  |
| Maternal 25(OH)D during pregnancy | -0.58 (-1.26;0.09) | 0.090 |  | -0.39 (-1.07;0.29) | 0.26 |
| Child’s 25(OH)D, 12 months | -0.22 (-0.67;0.23) | 0.33 |  | -0.28 (-0.73;0.17) | 0.22 |
| Child’s 25(OH)D, 24 months | 0.15 (-0.31;0.60) | 0.52 |  | -0.05 (-0.51;0.41) | 0.83 |
| ***Quadratic* *associations*** |  |  |  |  |  |
| Maternal 25(OH)D during pregnancy | -0.01 (-0.02;0.01) | 0.40 |  | -0.01 (-0.03;0.01) | 0.28 |
| Child’s 25(OH)D, 12 months | 0.00 (-0.01;0.01) | 0.48 |  | 0.00 (-0.00;0.01) | 0.34 |
| Child’s 25(OH)D, 24 months | 0.00 (-0.01;0.02) | 0.73 |  | 0.00 (-0.01;0.02) | 0.64 |

Model 1: crude, Model 2: Child’s sex, parent’s education, mother’s BMI, and season of birth controlled

Quadratic associations: Quadratic term added to the linear models, β: non-standardized, 25(OH)D=25-hydroxyvitamin D

| **Table 8** Associations between 25(OH)D concentration and indices of BRIEF. Change in indices per 10 nmol/l increase in 25(OH)D levels | | | | | |
| --- | --- | --- | --- | --- | --- |
|  | **Model 1** |  |  | **Model 2** |  |
|  | **β(95% CI)** | **p** |  | **β(95% CI)** | **p** |
| **Behavioral Regulation Index** |  |  |  |  |  |
| ***Linear associations*** | | | | | |
| Maternal 25(OH)D during pregnancy | -0.10 (-0.62;0.42) | 0.70 |  | -0.09 (-0.61;0.42) | 0.72 |
| Child’s 25(OH)D, 12 months | -0.36 (-0.73;0.01) | 0.056 |  | -0.27 (-0.63;0.08) | 0.13 |
| Child’s 25(OH)D, 24 months | -0.24 (-0.63;0.14) | 0.21 |  | -0.29 (-0.66;0.09) | 0.14 |
| ***Quadratic* *associations*** | | | | | |
| Maternal 25(OH)D during pregnancy | 0.01 (-0.00;0.02) | 0.12 |  | 0.01 (-0.01;0.02) | 0.36 |
| Child’s 25(OH)D, 12 months | -0.00 (-0.01;0.01) | 0.86 |  | 0.00 (-0.00;0.01) | 0.36 |
| Child’s 25(OH)D, 24 months | -0.00 (-0.01;0.01) | 0.90 |  | -0.00 (-0.01;0.01) | 0.97 |
| **Metacognition Index** |  |  |  |  |  |
| ***Linear associations*** | | | | | |
| Maternal 25(OH)D during pregnancy | -0.56 (-1.29;0.18) | 0.14 |  | -0.53 (-1.24;0.19) | 0.15 |
| Child’s 25(OH)D, 12 months | -0.50 (-0.10;0.02) | 0.060 |  | -0.43 (-0.93;0.07) | 0.089 |
| Child’s 25(OH)D, 24 months | 0.06 (-0.59;0.48) | 0.84 |  | -0.13 (-0.66; 0.39) | 0.61 |
| ***Quadratic* *associations*** | | | | | |
| Maternal 25(OH)D during pregnancy | 0.01 (-0.01;0.02) | 0.40 |  | 0.00 (-0.02;0.02) | 0.94 |
| Child’s 25(OH)D, 12 months | 0.00 (-0.02;0.01) | 0.52 |  | -0.00 (-0.01;0.01) | 0.80 |
| Child’s 25(OH)D, 24 months | 0.01 (-0.01;0.02) | 0.28 |  | 0.01 (-0.01;0.02) | 0.40 |

Model 1: crude, Model 2: Child’s sex, parent’s education, mother’s BMI, and season of birth controlled

Quadratic associations: Quadratic term added to the linear models, β: non-standardized, 25(OH)D=25-hydroxyvitamin D
